# MicroRNA-550a-3-5p controls the brain metastasis of lung cancer by directly targeting YAP1

**DOI:** 10.1186/s12935-021-02197-z

**Published:** 2021-09-16

**Authors:** Liang Wei, Guangxue Wang, Cheng Yang, Yanfei Zhang, Yiming Chen, Chunlong Zhong, Qinchuan Li

**Affiliations:** 1 Department of Neurosurgery, East Hospital, Tongji University School of Medicine, 150 Jimo Road, Shanghai, 200120 China; 2 Research Center for Translational Medicine, East Hospital, Tongji University School of Medicine, 150 Jimo Road, Shanghai, 200120 China; 3 Department of Cardiothoracic Surgery, East Hospital, Tongji University School of Medicine, 150 Jimo Road, Shanghai, 200120 China

**Keywords:** Lung cancer, Brain metastasis, HBMECs, miR-550a-3-5p, YAP1

## Abstract

**Background:**

This study aimed to explore the potential regulatory mechanisms of brain metastasis and to identify novel underlying targets of lung cancer with brain metastasis.

**Methods:**

Exosomes were isolated from the plasma of lung cancer patients with or without brain metastasis and low or high metastatic lung cancer cells, and small RNA from plasma-derived exosomes were sequenced. Differentially expressed miRNAs (DE-miRNAs) were identified. Human brain microvascular endothelial cells (HBMECs) were transfected with miR-550a-3-5p mimics or inhibitors and exosomes. Cell viability, migration, and apoptosis/cycle were determined using Cell Counting Kit-8 (CCK-8), Transwell, and flow cytometry, respectively. Western blotting was used to measure the expression of the associated proteins. Finally, a dual-luciferase reporter gene assay was performed to confirm the miR-550a-3-5p target.

**Results:**

Transmission electron microscopy, NanoSight, and western blotting showed that exosomes were successfully isolated and cell-derived exosomes could be taken up by HBMECs. Sequencing identified 22 DE-miRNAs which were enriched in the MAPK, chemokine, PPAR, and Wnt signaling pathways. MiR-550a-3-5p was significantly enriched in brain metastatic exosomes. Cellular experiments showed that miR-550a-3-5p and exosome enrichment significantly inhibited cell viability and migration, promoted apoptosis, and regulated the cell cycle of HBMECs compared with the controls (*P*  <  0.05). Compared with the controls, high levels of both miR-550a-3-5p and exosomes markedly upregulated cleaved-PARP expression, but downregulated the expression of pRB, CDK6, YAP1, CTGF, and CYR61 (*P*  <  0.05). Finally, YAP1 was confirmed to bind directly to miR-550a-3-5p.

**Conclusion:**

Our results indicate that miR-550a-3-5p and YAP1 may be novel potential targets for controlling brain metastasis.

**Supplementary Information:**

The online version contains supplementary material available at 10.1186/s12935-021-02197-z.

## Background

Lung cancer is the leading cause of cancer deaths worldwide, and can induce local and systemic immunosuppression and promote tumor growth and metastasis [1]. Brain metastasis is an important cause of morbidity and mortality in lung cancer patients [2]. At initial diagnosis, 10–15% of lung cancer patients are also diagnosed with brain metastasis and approximately 40% develop brain metastasis during their illness [3–5]. Treatment for patients diagnosed with early stage lung cancer usually involves surgical resection with occasional adjuvant radiotherapy or chemotherapy [6]. However, once patients develop lung cancer with brain metastasis, radical treatment becomes almost impossible, and the focus shifts to life-prolonging treatments, primarily systemic chemotherapy [1, 7]. Even after multimodal therapy, the survival of lung cancer patients with brain metastasis remains low, limited to weeks or months [4]. To date, the molecular mechanisms that can inhibit the progression of lung cancer metastases have not been identified, thus there is an urgent need to identify new targets for the prevention and control of brain metastasis of lung cancer.

Exosomes are membrane vesicles approximately 30–300 nm in diameter that are secreted by almost all cells [8]. Exosomes act as tools for cell-to-cell communication and contain RNAs, proteins, lipids, and other bioactive molecules, and are receiving increasing attention [9]. Studies have shown that the contents of cancer-derived exosomes can be transferred to other types of cells and pre-metastatic niches to regulate cell function and promote tumor progression [10–12]. Ham et al. [13] showed that breast cancer-associated exosomes induced the secretion of interleukin (IL)-6 and a survival promoting phenotype in macrophages through the glycoprotein 130-STAT3 signaling pathway. Additionally, an increasing body of evidence indicates that exosomal microRNAs (miRNAs) are involved in metastasis and can serve as potential targets in the treatment of brain metastasis [14, 15]. MiRNAs, a subgroup of non-coding RNAs that are approximately 22 nucleotides long, have been reported to participate in a variety of physiological and pathological processes such as cell proliferation, migration, apoptosis, invasion, and angiogenesis by regulating their target genes [16, 17]. A study by Zeng et al. [18] demonstrated that miR-25-3p (an miRNA that promotes colorectal cancer metastasis) was transferred to endothelial cells by colorectal cancer-derived exosomes and facilitated the formation of pre-metastatic niches by inducing vascular permeability and angiogenesis. Another study showed that breast cancer-associated fibroblast-derived exosomes significantly promoted tumor progression and damaged the function of tumors by infiltrating immune cells via the miR-92/PD-L1 pathway [19]. However, few key miRNAs that link lung cancer with brain metastasis have been reported, and their potential mechanisms in brain metastasis remain unknown.

Therefore, our study sequenced RNA from exosomes isolated from the plasma of lung cancer patients with or without brain metastasis and identified differentially expressed miRNAs (DE-miRNAs). miR-550a-3-5p was significantly upregulated in exosomes isolated from the plasma of lung cancer patients with brain metastasis, and was selected for further cell mechanism studies. Our findings improve understanding of the occurrence and progression of brain metastases in lung cancer patients and identified new potential therapeutic targets and pathways underlying brain metastasis.

## Methods

### Collection of clinical samples and isolation of exosomes from plasma

This study was approved by the Human Biomedical Research Ethics committee of Shanghai East Hospital affiliated to Tongji University [(2020) Research on No.135], and informed consent was obtained from all participants. Lung cancer patients with brain metastasis (n  =  3) and lung cancer patients without brain metastasis (n  =  3) were recruited from Shanghai East Hospital affiliated with Tongji University, and a 10 mL blood sample was collected from each patient. Next, exosomes were isolated from the plasma at 4 °C. Five millilitre plasma was then centrifuged at 12,000×*g* for 30 min and the supernatant transferred to a new tube. After filtration through a 0.22 μm sterile filter, the supernatant was centrifuged at 1,20,000×*g* for 60 min. The pellet was resuspended in pre-cooled PBS and transferred to a clean ultra-high-speed centrifuge tube. After centrifugation at 1,20,000×*g* for 60 min, the pellet was resuspended in 200 μL pre-cooled PBS and stored at − 80 °C.

### Cell culture and isolation of exosomes from 95C and 95D cell lines

Human low metastatic lung cancer cell line 95C, human high metastatic lung cancer cell line 95D, and human brain microvascular endothelial cells (HBMECs) were purchased from Cell Bank, Chinese Academy of Sciences (Shanghai, China). 95C and 95D cell lines were cultured in Dulbecco’s modified Eagle’s medium (DMEM; Gibco, Grand Island, NY, USA) containing 10% fetal calf serum (FBS, Gibco) and 1% penicillin/streptomycin (Gibco) and incubated at 37 °C in an incubator with 5% CO_2_. HBMECs were maintained in F12K medium (Gibco) with 10% FBS and 1% penicillin/streptomycin and then cultured at 37 °C in an incubator with 5% CO_2_. The cells were passaged once they reached 80–90% confluence.

Exosomes were extracted from the 95C and 95D cells at 4 °C. Briefly, the 95C and 95D cells were harvested and washed with PBS. The cells were cultured for 48 h in serum-free medium and then transferred to a 50 mL tube and centrifuged at 5000×*g* for 5 min. The supernatant was collected and centrifuged again at 2000×*g* for 30 min, and the supernatant collected. Next, an equal volume of pre-cooled 16% PEG 6000 was added to, and thoroughly mixed, with the supernatant. After overnight incubation at 4 °C, the mixture was centrifuged at 10,000×*g* for 60 min, the pellet collected and resuspended in 1 mL PBS. After centrifuged at 1,00,000×*g* for 70 min, the pellet was resuspended in 200 μL PBS and stored at − 80 °C until use.

### Identification of exosomes

The concentration of isolated exosomes was determined using a BCA assay kit (BOSTER Biological Technology Co., Ltd., CA, USA), following the manufacturer’s instructions. Thereafter, a transmission electron microscope (TEM, JEOL LTD, Peabody, MA, USA) was used to visualize the morphology of exosomes, as previously described [20]. Following the protocols described by Soares et al. [21], particle exosome sizes were measured using a NanoSight NS300 particle size analyzer (NTA, Malvern Panalytical, Malvern, UK). Western blotting was used to determine the expression of exosome-specific protein markers, including CD9, CD63, and HSP70 [22].

### Small RNA sequencing of plasma-derived exosomes

The plasma-derived exosomes were sent to Yanzai Biotechnology (Shanghai) Co., Ltd., (Shanghai, China) for small RNA sequencing. The Expdiff method was used to identify differentially expressed miRNAs (DE-miRNAs) between lung cancer patients with brain metastasis and lung cancer patients without brain metastasis. The thresholds for screening DE-miRNAs were |log2Fold change (FC)|>  1 and P  <  0.05. Targetscan, miRanda, and PITA software were used to predict the target genes of the DE-miRNAs [23]. These DE-miRNAs were then submitted for Gene Ontology (GO) and Kyoto Encyclopedia of Genes and Genomes (KEGG) pathway analyses. Enriched GO terms and KEGG pathways with P  <  0.05 were identified.

### Quantitative reverse transcription PCR (RT-qPCR)

We selected five DE-miRNAs (three upregulated and two downregulated) for further validation. Total RNA was isolated from plasma-derived exosomes using RNAiso Plus kit (Trizol, Takara Biomedical Technology Co., Ltd., Beijing, China) and twice the volume of isopropanol, following the manufacturer’s recommendations. The levels of miRNAs in the exosomes were determined using the stem ring method [24]. Briefly, total RNA was reverse transcribed into cDNA using the PrimeScript™ II 1st Strand cDNA synthesis Kit (Takara Biomedical Technology Co., Ltd.). First, 20 μL denaturing reaction solution, including 3 μL RT primer (10 μM), dNTP mixture (10 nM), RNA template (10 μL), and RNase free dH_2_O (6 μL), was prepared and used for miRNA reverse transcription. The total volume for miRNA reverse transcription was 20 μL and included 14 μL denaturing reaction solution, 4 μL of 5  ×  PrimeScript II buffer, 0.5 μL RNase inhibitor (40 U/μL), 1 μL PrimeScript II RTase (200 U/μL), and 0.5 μL RNase free dH_2_O. miRNA reverse transcription was conducted at 42 °C for 60 min, followed by 95 °C for 5 min. Reverse transcriptase was used for qPCR. The total volume of qPCR was 20 µL and included 10 µL SYBR Premix EX Taq, 1 µL forward primer (10 µM), 1 µL reverse primer (10 µM), 2 µL cDNA, and 6 µL RNase free water. The RT-qPCR reaction was initiated at 50 °C for 2 min, followed by 40 cycles of 95 °C for 2 min, 95 °C for 15 s, and 60 °C for 60 s. The sequences of all primers are listed in Table 1. U6 served as a housekeeping gene, and the levels of miR-550a-3-5p, miR-1226-3p, miR-144-3p, miR-27a-3p, and miR-3200-5p in exosomes were calculated using the 2^−ΔΔCt^ method [25].

**Table 1:** The sequences of all primers

Primer	Sequence (5′–3′)
has-miR-550a-3-5p	JH:GTCGTATCCAGTGCAGGGTCCGAGGTATTCGCACTGGATACGACCTCTTA
	F: GCGCAGTGCCTGAGGGAG
hsa-miR-1226-3p	JH:GTCGTATCCAGTGCAGGGTCCGAGGTATTCGCACTGGATACGACCTAGGG
	F: GCCTCACCAGCCCTGTGTT
hsa-miR-3200-5p	JH:GTCGTATCCAGTGCAGGGTCCGAGGTATTCGCACTGGATACGACACCTTG
	F: GCGCAATCTGAGAAGGCGCA
hsa-miR-144-3p	JH:GTCGTATCCAGTGCAGGGTCCGAGGTATTCGCACTGGATACGACAGTACA
	F: GCGCGCGCTACAGTATAGATGA
hsa-miR-27a-3p	JH:GTCGTATCCAGTGCAGGGTCCGAGGTATTCGCACTGGATACGAC GCGGAA
	F: GCGCGTTCACAGTGGCTAAG
Downstream universal primer	R: GTGCAGGGTCCGAGGT
U6	RT:GTCGTATCCAGTGCAGGGTCCGAGGTATTCGCACTGGATACGACAAAATATG
	F: CTCGCTTCGGCAGCACA
	R: AACGCTTCACGAATTTGCGT

### Cellular uptake of cancer cell-derived exosomes in HBMECs

Lung cancer cell-derived exosomes were labeled with PKH67 (green fluorescent cell linker for general cell membrane labeling) using a commercial kit (PKH67GL-1KT; Sigma-Aldrich, USA) following the manufacturer’s instructions. Briefly, 700 μL lung cancer cell-derived exosomes and 1300 μL Diluent C were mixed, and then 16 μL PKH67 dye and 2 mL Diluent C were added. The mixture was incubated at room temperature for 5 min and 4 mL of 1% BSA was added to bind excess dye. After centrifugation at 1,20,000×*g* for 90 min at 4 °C, the pellet (PKH67-labeled exosomes) was resuspended in 300 μL PBS.

HBMECs were seeded in a 24-well plate and cultured overnight. Next, 10 μL PKH67-labeled exosomes were added to the cells. After culturing for 24 h and 48 h, the medium was removed and the cells washed with PBS. The HBMECs were then fixed with 4% paraformaldehyde at room temperature for 20 min. After washing, 0.1% TritonX-100 was added and the cells cultured for 20 min. After washing, 3% BSA was added and the mixture incubated for 1 h to block non-specific binding. After washing, mounting medium with DAPI was added to stain the cells and a laser scanning confocal microscope (TCS SP8, Leica Microsystems, Inc., USA) was used to observe the cells at 400  ×  magnification.

### Cell transfection

Cell transfection was carried out using the methods described by Wan et al. [26]. Briefly, HBMECs were seeded in a 24-well plate and cultured overnight. After the cells had grown to a density of 70%, the cells were transfected with 50 nM miR-550a-3-5p mimics, miR-550a-3-5p inhibitor, and miRNA negative control (NC) using Lipofectamine 2000 (Thermo Fisher Scientific), according to the manufacturer’s protocols. miR-550a-3-5p mimics, miR-550a-3-5p inhibitor, and miRNA NC were designed and provided by Yanzai Biotechnology Co., Ltd., (Shanghai, China). After 6 h of transfection, the medium was replaced with complete medium containing 10% FBS. After culturing for another 48 h, total RNA was extracted from the cells with different treatments, and the level of miR-550a-3-5p was measured using RT-qPCR to evaluate the cell transfection efficiency. The miR-550a-3-5p sequences are shown in Table 1.

### Cell viability and migration assays

HBMECs were seeded in a 96-well plate and divided into five groups: control, miRNA NC, miR-550a-3-5p mimics, miR-550a-3-5p inhibitor, and exosomes. The cells in the miRNA NC, miR-550a-3-5p mimics, miR-550a-3-5p inhibitor, and exosomes were transfected with miRNA NC, miR-550a-3-5p mimics, miR-550a-3-5p inhibitor, and high metastatic lung cancer cell-derived exosomes (final concentration of 10 μg/mL), respectively. The cells in the control group were treated with an equal volume of PBS. After culturing for 24 h, 48 h, 72 h or 96 h, 10 μL of cell counting kit-8 (CCK-8, Beyotime Biotechnology) was added to the cells and cultured for a further 2 h, The optical density was measured at 450 nm using a microplate reader (Thermo Fisher Scientific).

Migration of HBMECs was assessed using Transwell chambers (pore size 8 μm; Guangzhou Jet Bio-Filtration Co., Ltd., Guangzhou, China). After the different treatments, HBMECs were harvested and resuspended in serum-free medium to adjust the cell density to 3  ×  10^5^ cells/mL. Thereafter, 0.2 mL cell suspensions were inoculated into the upper chamber of Transwell chambers and complete medium containing 10% FBS was added into the lower chamber. After culturing for 24 h, the Transwell chambers were removed and washed twice with PBS. The cells were fixed with 4% paraformaldehyde for 10 min, washed twice with PBS, stained with 0.5% crystal violet (Beyotime Biotechnology), and then incubated at room temperature for 10 min. After washing, the cells were observed and photographed under a microscope.

### Cell apoptosis and cycle assays

The apoptosis of HBMECs subjected to different treatments was determined using an Annexin V-FITC/PI apoptosis assay kit, according to the manufacturer’s protocol. Briefly, cells subjected to different treatments were collected and resuspended in 100 μL of 1  ×  binding buffer. Subsequently, 5 μL FITC-Annexin V and 5 μL PI (50 μg/mL) were added and the mixture incubated in the dark at 25 °C for 15 min. Finally, 400 μL of 1  ×  binding buffer was added and cell images acquired using flow cytometry.

The cell cycle was also detected using flow cytometry. Cells in each treatment group were centrifuged at 1000 rpm for 5 min and resuspended in 0.5 mL PBS. Next, 5 mL of 70% pre-cooled ethyl alcohol was added to the cells and the mixture incubated overnight at 4 °C. The next day, the cells were centrifuged at 1500 rpm for 6 min and washed with 6 mL PBS. After centrifugation at 1000 rpm for 6 min, the pellet was resuspended in 0.3 mL PBS and transferred to a new tube. RNase A was added and after incubation at 37 °C for 30 min, 5 μL PI was added. After staining in the dark at 4 °C for 15 min, a flow cytometer was used to determine the cell cycle.

### Western blot assays

Total protein was isolated from cells subjected to different treatments using RIPA lysis buffer (Beyotime Biotechnology) and the concentrations of total protein were determined using a BCA assay kit (BOSTER Biological Technology Co., Ltd.). Protein samples (20 μg) were separated using 10% SDS-PAGE and then transferred to PVDF membranes. After blocking with 5% skim milk, the membranes were incubated with anti-cleaved-Poly-(ADP-ribose) polymerase (PARP) antibody (1:1000, Abcam), anti-RB transcriptional corepressor 1 (pRB) antibody (1:1000, Abcam), anti-cyclin dependent kinase 6 (CDK6) antibody (1:1000, Abcam), anti-Yes1 associated transcriptional regulator (YAP1) antibody (1:1000, Abcam), anti- connective tissue growth factor (CTGF) antibody (1:1000, Proteintech Group, Inc.), anti-cysteine rich angiogenic inducer 61 (CYR61) antibody (1:1000, Abcam), and anti-GAPDH antibody (1:10,000, Proteintech Group, Inc.) overnight at 4 °C. After washing three times with PBST (PBS with 0.05% Tween20), the membranes were incubated with the secondary antibody (1: 5000, Jackson ImmunoResearch) at 37 °C for 2 h. After five washes with PBST, the protein bands were visualized using a Millipore ECL system (Shanghai Tanon Technology Co. Ltd., Shanghai, China).

### Dual-luciferase reporter gene assay

Choe et al. [27] previously showed that *YAP1* is the target gene of miR-550a-3-5p in human colon cancer cells (HCT116). A dual-luciferase reporter gene assay was carried out to further verify the conclusion in the lung cancer cell-derived exosomes. The sequence of the YAP1 3′-untranslated region (3′-UTR) was designed and provided by Yanzai Biotechnology Co., Ltd., (Shanghai, China) and the pGL3-basic plasmid was used to construct the YAP1 3′-UTR reporter plasmid (pGL3-YAP1). 293 T cells were then seeded in a 96-well plate at a density of 4  ×  10^4^ cells/well and cultured overnight. The next day, the medium was changed to serum-free medium, and 0.4 μg pGL3-basic plasmid or 0.4 μg pGL3-YAP1 were co-transfected into 293 T cells with 100 nM miR-550a-3-5p mimics or 100 nM NC mimics using Lipofectamine 3000 (Thermo Fisher Scientific, Waltham, MA, USA) following the manufacturer’s instructions. After 6 h of transfection, the cell medium was replaced with complete medium. After culturing for another 48 h, the cell supernatant was removed and 150 μL of lysis buffer from the dal-luciferase reporter gene assay kit (Beyotime Biotechnology) was added. After centrifugation at 12,000×*g* for 5 min, the supernatant was used to determine the relative light unit using a dal-luciferase reporter gene assay kit following the manufacturer’s recommendations.

### Statistical analysis

Graphpad prism 5 (Graphpad Software, San Diego, CA) was used for statistical analyses. Data are expressed as mean  ±  standard deviation (SD). For comparison between two groups, student’s t test was performed, whereas one-way analysis of variance (ANOVA) followed by Bonferroni method was used to compare significant differences among more than two groups. *P*  <  0.05 was considered statistically significant.

## Results

### Characterization of plasma-derived exosomes and lung cancer cell-derived exosomes

**Fig. 1:**
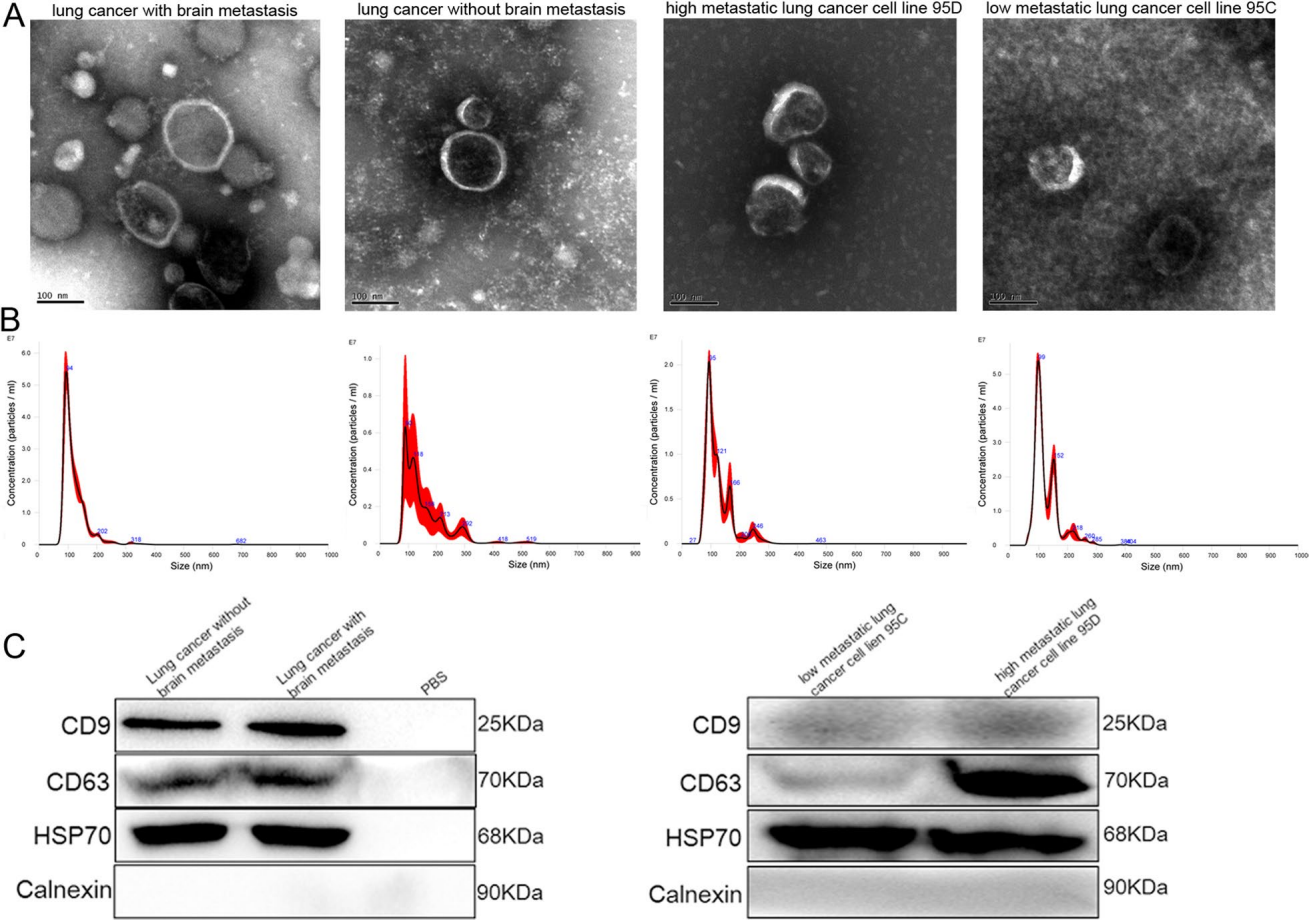
Characterization of exosomes isolated from plasma of lung cancer patients with or without brain metastasis and high or low metastatic lung cancer cells. **A** Transmission electron microscopy was used to visualize the morphology of exosomes. **B** The particle size of exosomes was measured using a NanoSight NS300 particle size analyser. **C** The levels of CD9, CD63, and HSP70 protein were determined through western blotting

Exosomes were extracted from the plasma of lung cancer patients with or without brain metastasis and high or low metastatic lung cancer cells, and characterized using NTA, TEM, and western blotting. TEM results showed that the exosomes were cup-shaped or nearly round and approximately 100 nm in diameter (Fig. 1A). NTA results showed that the major peaks of substances isolated from the plasma of lung cancer patients with or without brain metastasis and high or low metastatic lung cancer cells were approximately 94 nm or 90 nm and 95 nm or 99 nm, respectively (Fig. 1B). These results are in agreement with the size distribution of exosomes, as previously reported [28]. Finally, western blotting showed that CD9, CD63, and HSP70, which are exosome markers, were all expressed in the exosomes, while calnexin was not (Fig. 1C). These results indicate that exosomes were successfully isolated from the plasma and cells.

### Small RNA sequence analyses and RT-qPCR validation

**Fig. 2:**
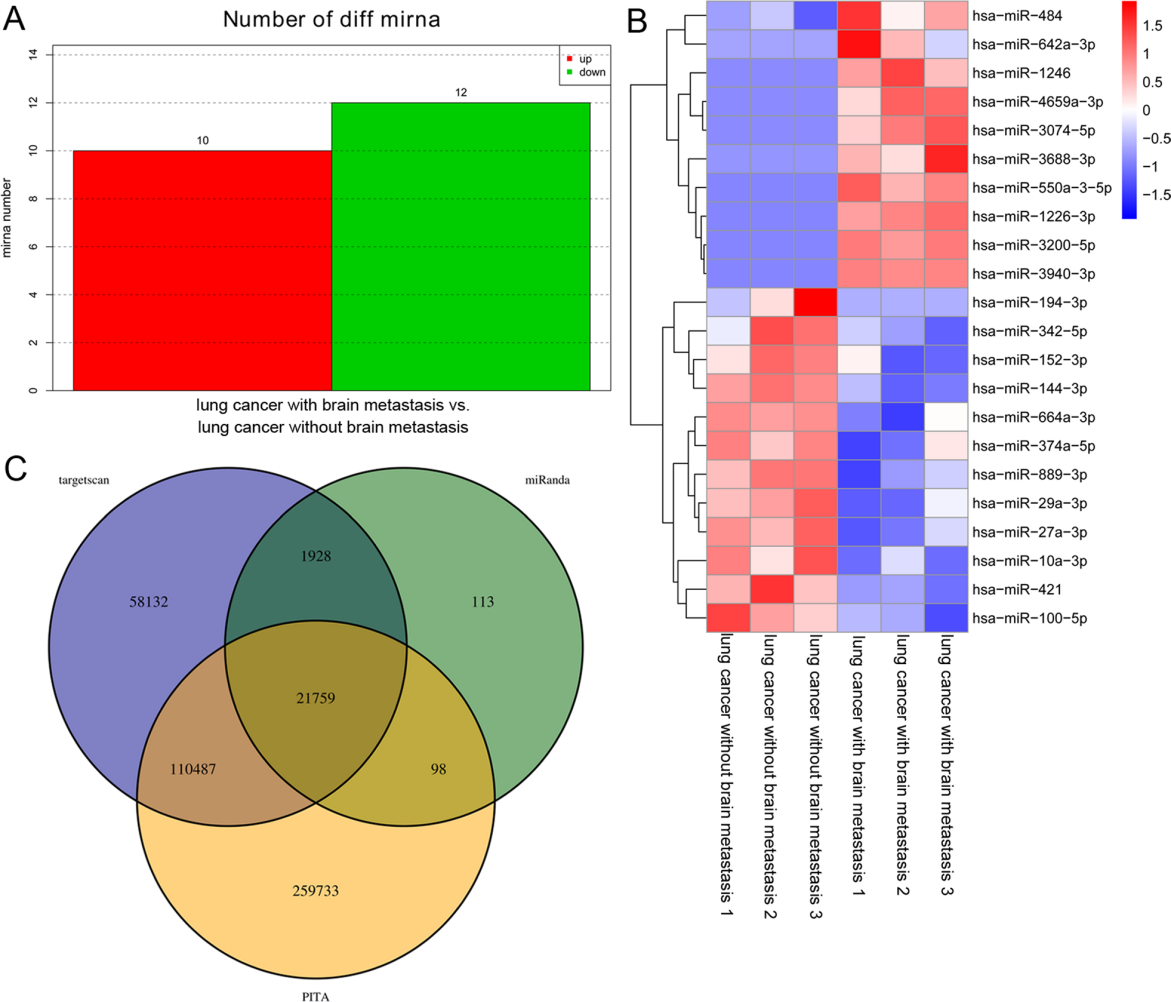
Small sequencing analyses of exosomes isolated from plasma of lung cancer patients with or without brain metastasis. **A** Differentially expressed miRNAs (DE-miRNAs) were identified based on the criteria: |log_2_ FC|> 1, and P  <  0.05. **B** Heatmap of the identified DE-miRNAs. **C** Venn diagram of target genes of the identified DE-miRNAs predicted by targetscan, miRanda, and PITA software

Exosomes isolated from the plasma of lung cancer patients with or without brain metastasis were sequenced. A total of 22 DE-miRNAs, including 10 upregulated and 12 downregulated miRNAs, were identified based on the criteria: |log_2_ FC|>  1 and P  <  0.05, (Fig. 2A). A heatmap representing the cluster analysis of these DE-miRNAs is shown in Fig. 2B. A total of 21,759 mRNA targets of these DE-miRNAs were predicted using TargetScan, miRanda, and PITA software (Fig. 2C). The DE-miRNAs were then subjected to GO term and KEGG pathway analyses. A total of 44 biological processes (BP), 9 cellular components (CC), and 13 molecular functions (MF) with P  <  0.05 were obtained, including anatomical structure development, biological adhesion, biological regulation, and cell communication in BP; cell periphery, extracellular matrix, and plasma membrane in CC; and DNA binding, phosphatase activity, signal transducer activity, and transmembrane transporter activity in MF (Additional file 1: Figure S1A). The DE-miRNAs were significantly enriched in the MAPK signaling pathway, oxytocin signaling pathway, axon guidance, neuroactive ligand-receptor interaction, calcium signaling pathway, chemokine signaling pathway, PPAR signaling pathway, Wnt signaling pathway, and retinol metabolism (Additional file 1: Figure S1B).

**Fig. 3:**
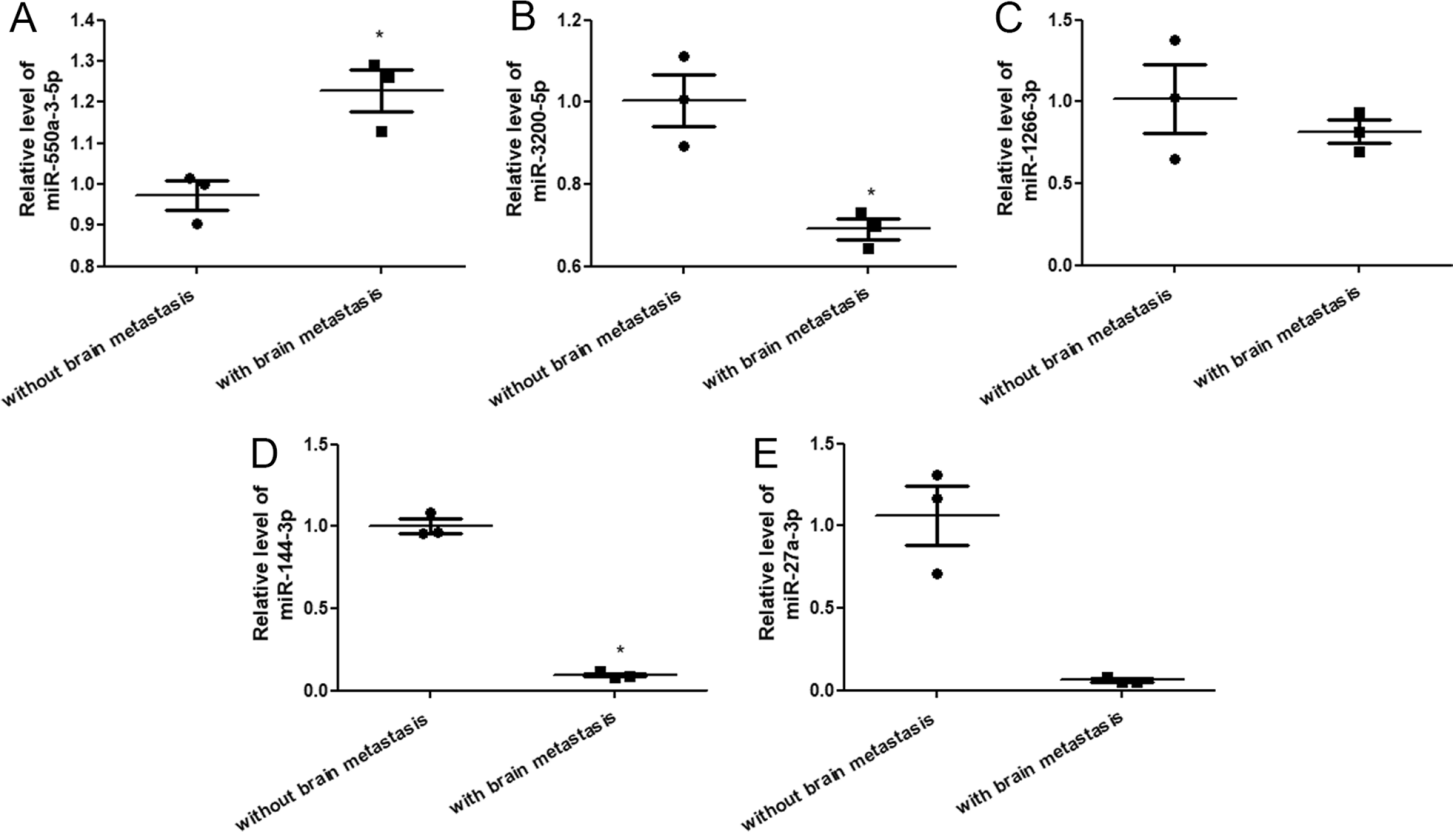
Verification of sequencing data. The levels of miR-550a-3-5p (**A**), miR-3200-5p (**B**), miR-1226-3p (**C**), miR-144-3p (**D**) and miR-27a-3p (**E**) in exosomes isolated from plasma of lung cancer patients with or without brain metastasis. *P  <  0.05, compared with exosomes isolated from plasma of lung cancer patients without brain metastasis

Five DE-miRNAs, including three upregulated miRNAs (hsa-miR-550a-3-5p, hsa-miR-1226-3p, and hsa-miR-3200-5p) and two downregulated miRNAs (hsa-miR-144-3p and hsa-miR-27a-3p), were selected and their levels in the different plasma-derived exosomes determined using RT-qPCR. miR-550a-3-5p levels in the exosomes isolated from lung cancer patients with brain metastasis were significantly higher than those in the exosomes isolated from lung cancer patients without brain metastasis (*P*  <  0.05, Fig. 3A). However, the miR-3200-5p levels in the exosomes isolated from lung cancer patients with brain metastasis were opposite to those of miR-550a-3-5p (Fig. 3B). No significant differences in miR-1266-3p levels were observed between exosomes isolated from lung cancer patients with brain metastasis and lung cancer patients without brain metastasis (*P*  >  0.05; Fig. 3C). The levels of miR-144-3p and miR-27a-3p in the exosomes isolated from lung cancer patients with brain metastasis were clearly decreased compared with those in the exosomes isolated from lung cancer patients without brain metastasis (*P * <  0.05; Fig. 3D, E). These results indicate that the consistency rate between RT-qPCR and sequencing was 60%, which implies a relatively high reliability of the sequencing results. Therefore, we selected hsa-miR-550a-3p for subsequent experiments.

### Cellular uptake of lung cancer cell-derived exosomes and cell transfection efficiency analysis

**Fig. 4:**
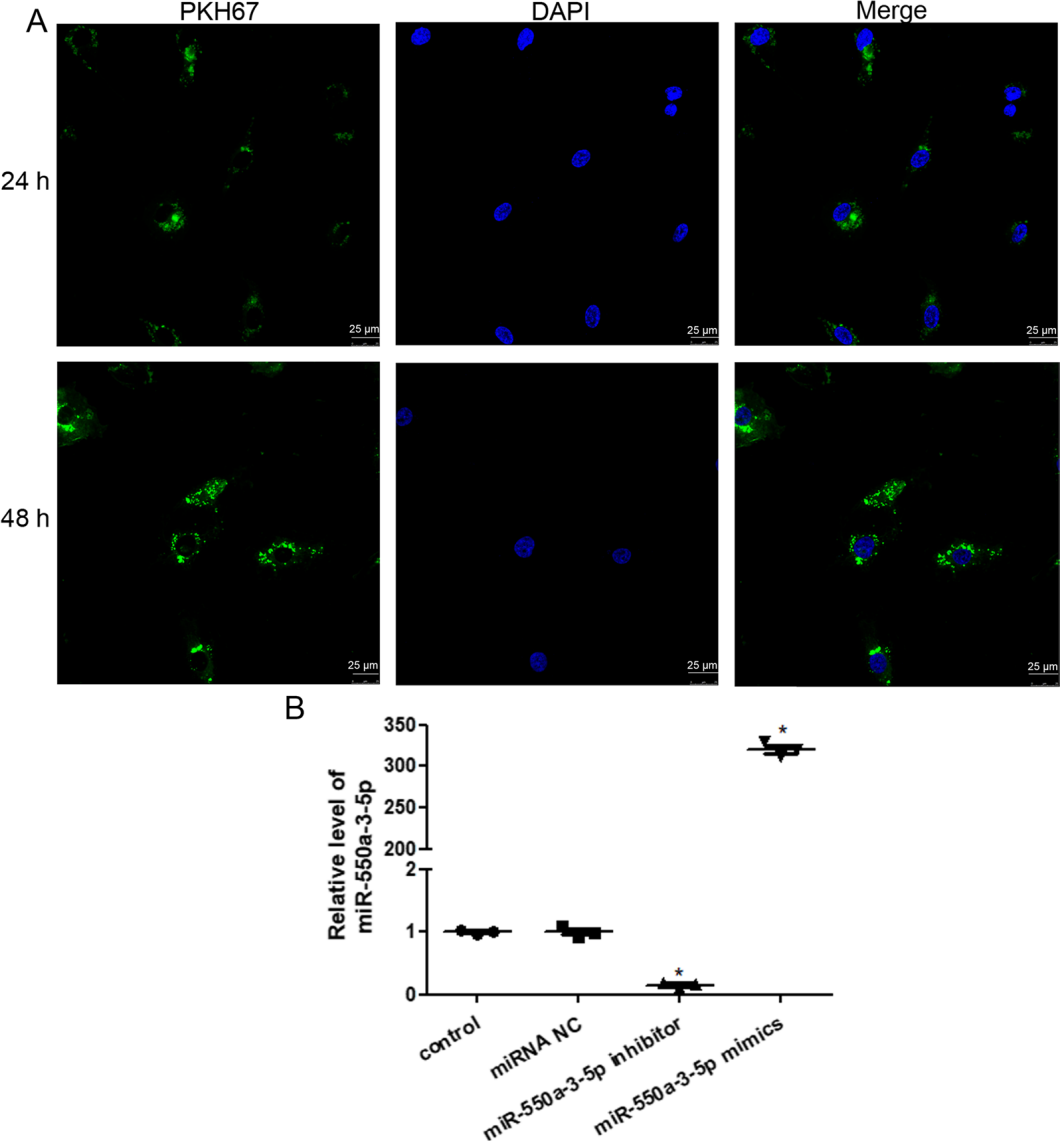
Cellular uptake of lung cancer cell-derived exosomes and cell transfection efficiency analysis. **A** Cell-derived exosomes were labeled green by PKH67 and PKH67-labeled exosomes could be taken up by human brain microvascular epithelial cells (HBMECs) after co-culturing. **B** The relative levels of miR-550a-3-5p after transfection with miR-550a-3-5p inhibitor and mimics. *P  <  0.05, compared with the control group

Lung cancer cell-derived exosomes were labeled with green fluorescence through PKH67 staining and HMBECs were stained blue by DAPI. After co-culturing for 24 h or 48 h, HBMECs took up PKH67-labeled exosomes, and the green fluorescence was stronger after 48 h of co-culturing compared with 24 h of co-culturing (Fig. 4A). The results indicate that lung cancer cell-derived exosomes can be taken up by HBMECs, with the fluorescence intensity increasing with the increase in culturing time.

Next, to construct HBMECs with miR-550-3-5p overexpression and knockdown, miR-550a-3-5p mimics and inhibitor were transfected into HBMECs. No significant difference was observed in miR-550a-3-5p levels between the control and miRNA NC groups. Compared with the control group, miR-550a-3-5p levels were significantly decreased in the miR-550a-3-5p inhibitor group (*P * <  0.05) but significantly increased in the miR-550a-3-5p mimic group (*P*  <  0.05; Fig. 4B). These results indicate that HBMECs with miR-550-3-5p overexpression and knockdown were successfully established and could be used for further experiments.

### Cell viability and migration analyses

**Fig. 5:**
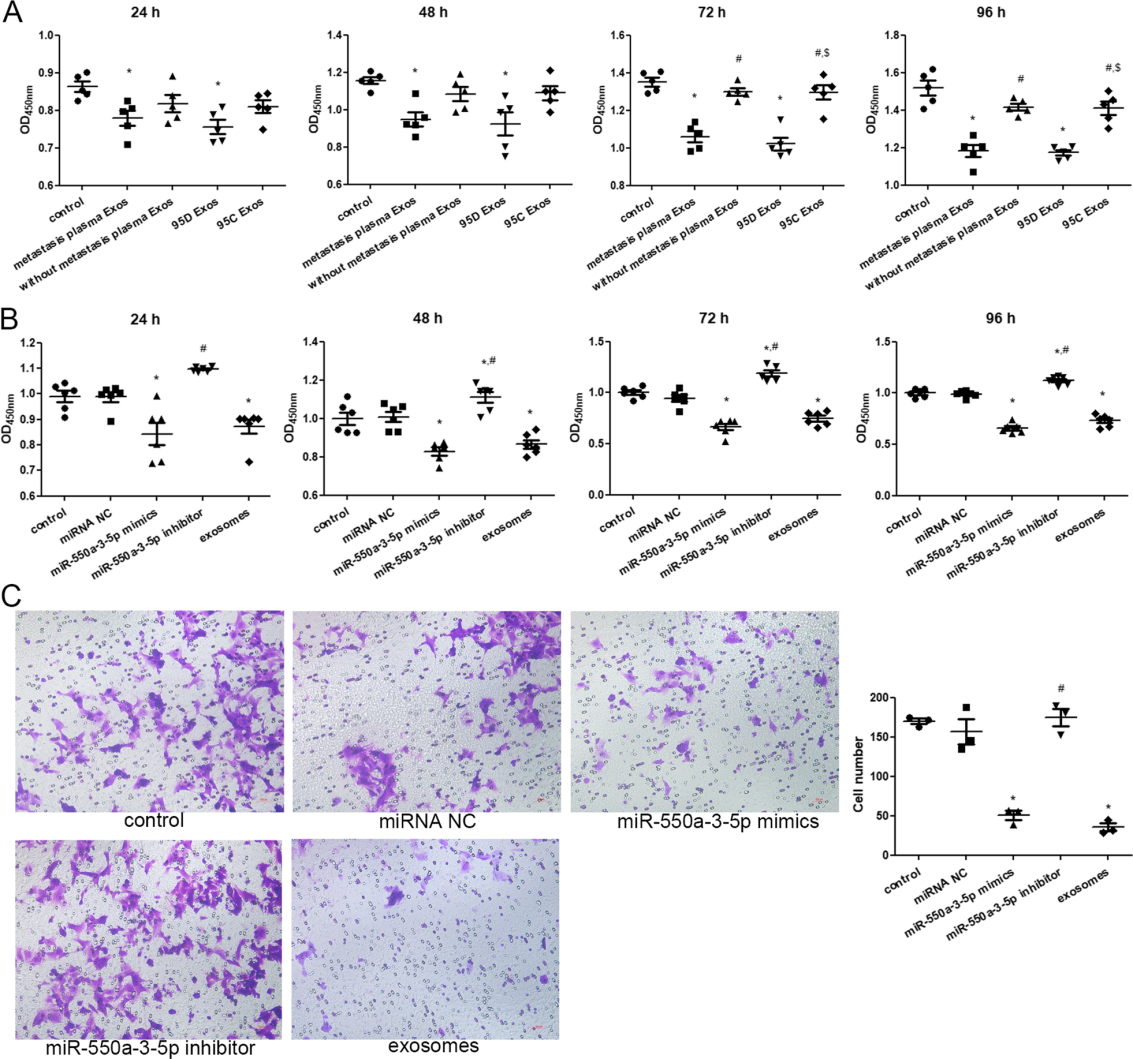
The effects of miR-550a-3-5p on cell viability and migration. **A** The viability of HBMECs treated with different exosomes was measured using Cell Counting Kit-8 (CCK-8) after 24 h, 48 h, 72 h, and 96 h of culturing. *P  <  0.05, compared with the control group; ^#^*P * <  0.05, compared with the lung cancer with brain metastasis plasma-derived exosomes group. ^$^*P*  <  0.05, compared with 95D cells-derived exosomes group. **B** Viability of HBMECs with different transfections was determined using CCK-8 after 24, 48, 72 and 96 h of culturing. **C** Migration of HBMECs with different transfections was measured using Transwell assay. Left: the images of crystal violet staining. Right: cell numbers in the different groups. *P  <  0.05, compared with the control group; ^#^*P*  <  0.05, compared with the miR-550a-3-5p mimics group

The effect of miR-550a-3-5p on the viability of HBMECs was assessed using CCK-8. First, the exosomes isolated from different plasma and cells were treated with HBMECs for 24 h, 48 h, 72 h, and 96 h. There was no significant difference in cell viability among control, lung cancer without brain metastasis plasma-derived exosomes, and 95C (low metastatic lung cancer cells)-derived exosomes (Fig. 5A). After culturing for 24 h, 48 h, 72 h, and 96 h, the viability of HBMECs treated with lung cancer with brain metastasis plasma-derived exosomes (higher miR-550a-3-5p levels) and 95D (high metastatic lung cancer cells)-derived exosomes (higher miR-550a-3-5p levels) was significantly lower than that of the control group (*P * <  0.05; Fig. 5A). These results indicate that exosomes with higher miR-550a-3-5p levels can inhibit the viability of HBMECs.

As shown in Fig. 5B, no significant difference in cell viability was observed between the control and miRNA NC groups (*P * >  0.05). After cultured for 24 h, cell viability was significantly decreased in the miR-550a-3-5p mimics and exosomes groups compared with the control group (*P * <  0.05), while cell viability was significantly higher in the miR-550a-3-5p inhibitor group than in the miR-550a-3-5p mimics group (*P * <  0.05; Fig. 5B). After culturing for 48 h, 72 h and 96 h, miR-550a-3-5p and exosomes significantly reduced cell viability (*P * <  0.05), whereas miR-550a-3-5p inhibitor significantly enhanced cell viability compared with the control (*P*  <  0.05; Fig. 5B). These results indicate that miR-550a-3-5p enrichment, similar with lung cancer with brain metastasis plasma-derived exosomes and high metastatic lung cancer cell-derived exosomes, could reduce the viability of HBMECs, while miR-550a-3-5p inhibition had the opposite effect.

Cell migration was determined using the Transwell assay. There was no significant difference in cell migration between control and miRNA NC groups, or between miR-550a-3-5p mimics and exosome groups (*P * >  0.05; Fig. 5C). After transfection with miR-550a-3-5p mimics and exosomes, the cell numbers were significantly decreased compared with the controls (*P*  <  0.05); whereas the cell numbers in the miR-550a-3-5p inhibitor group was significantly higher than in the miR-550a-3-5p mimics group (*P * <  0.05; Fig. 5C). These results suggest that miR-550a-3-5p enrichment and high metastatic lung cancer cell-derived exosomes can inhibit the migration of HBMECs.

### Apoptosis and cell cycle analysis

**Fig. 6:**
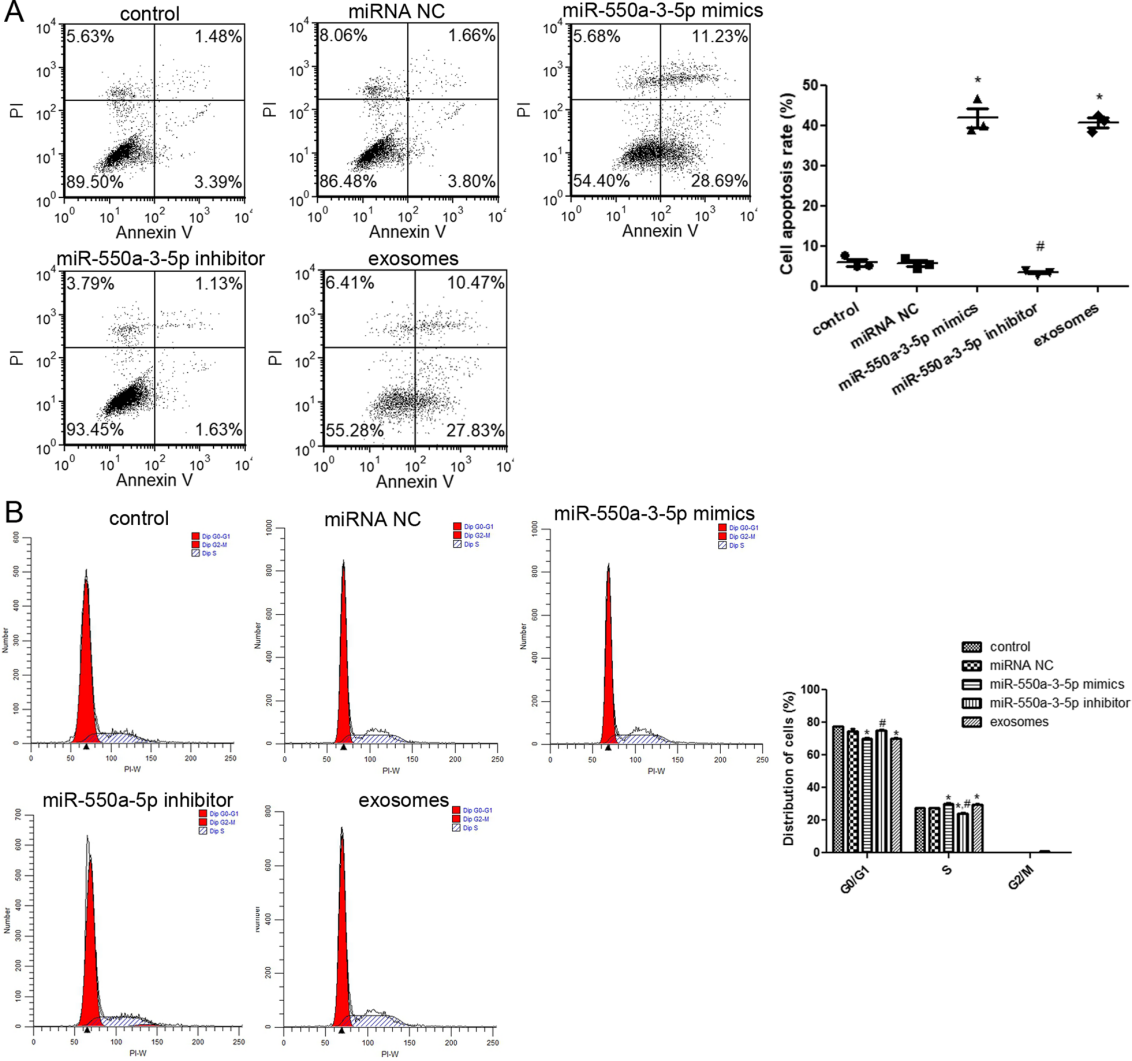
The effects of miR-550a-3-5p on apoptosis and cell cycle. **A** Apoptosis of HBMECs with different transfections was determined. Left: images acquired by flow cytometry. Right: cell apoptosis rates in the different groups. **B** Cell cycle of HBMECs with different transfections was detected using flow cytometry. *P  <  0.05, compared with the control group; ^#^*P * <  0.05, compared with the miR-550a-3-5p mimics group

Flow cytometry was used to determine the effects of miR-550a-3-5p and lung cancer cell-derived exosomes on apoptosis and cell cycle of HBMECs. There was no significant difference in cell apoptosis between the control and miRNA NC groups (*P * >  0.05; Fig. 6A). Compared with the control group, cell apoptosis was significantly increased in the miR-550a-3-5p mimics and cell-derived exosome groups (*P * <  0.05), but was markedly decreased in the miR-550a-3-5p inhibitor group (*P * <  0.05; Fig. 6A). In addition, cell cycle analysis showed that the cells transfected with miR-550a-3-5p mimics and cell-derived exosomes were significantly reduced during the G0/G1 phase compared with controls (*P * <  0.05), whereas the number of cells in the miR-550a-3-5p inhibitor group was notably higher than in the miR-550a-3-5p mimic group (*P * <  0.05; Fig. 6B). During the S phase, miR-550a-3-5p mimics and cell-derived exosomes significantly increased the number of cells compared with controls (*P * <  0.05), and miR-550a-3-5p inhibitor significantly decreased the number of cells compared with the control and miR-550a-3-5p mimics (*P*  <  0.05; Fig. 6B). No significant differences were observed in the G2/M phase. These results indicate that miR-550a-3-5p enrichment, similar with high metastatic lung cancer cell-derived exosomes, could promote apoptosis of HBMECs and regulate cell distribution in the G0/G1 and S phases.

### Western blot assays

**Fig. 7:**
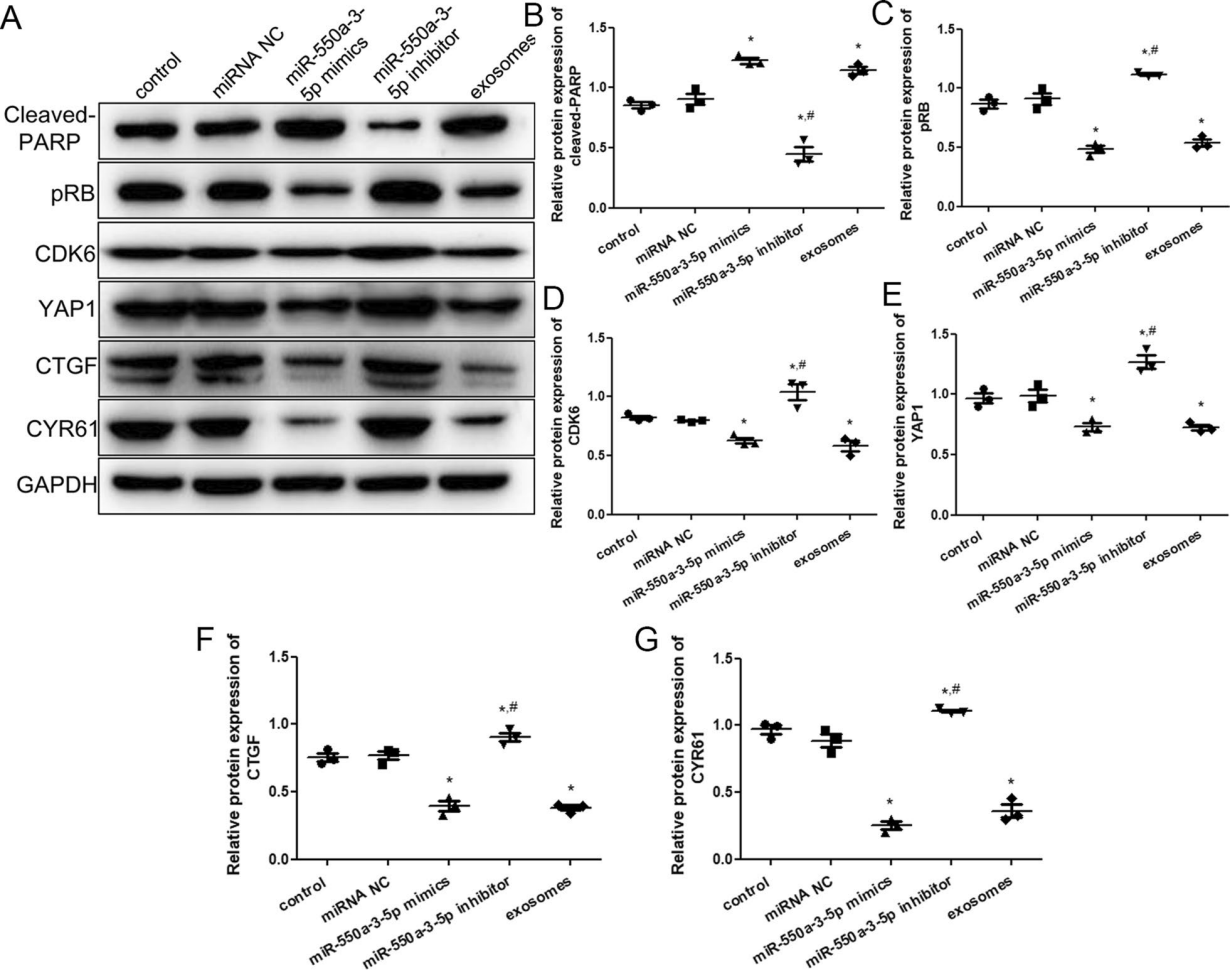
The effects of miR-550a-3-5p on cleaved-PARP, pRB, CDK6, YAP1, CTGF, and CYR61. **A** Western blotting images. **B** Cleaved-PARP protein expression. **C** pRB protein expression. **D** CDK6 protein expression. **E** YAP1 protein expression. **F** CTGF protein expression. (**G**) CYR61 protein expression. *P  <  0.05, compared with the control group; ^#^*P * <  0.05, compared with the miR-550a-3-5p mimics group

The expression levels of cleaved-PARP, pRB, CDK6, YAP1, CTGF, and CYR61 proteins were measured using western blotting. The bands corresponding to the specific proteins are shown in Fig. 7A. No significant differences were observed in the expression of cleaved-PARP, pRB, CDK6, YAP1, CTGF, and CYR61 proteins between control and miRNA NC groups, and between miR-550a-3-5p mimics and exosomes groups (*P * >  0.05; Fig. 7). Compared with the control group, the expression of cleaved-PARP was significantly upregulated in the miR-550a-3-5p mimic and exosome groups (*P*  <  0.05), but downregulated in the miR-550a-3-5p inhibitor group (*P*  <  0.05; Fig. 7A, B). However, the trend in the expression of pRB protein in the different groups was opposite to that of cleaved-PARP (Fig. 7A, C). The expression of CDK6, YAP1, CTGF, and CYR61 proteins was significantly lower in the miR-550a-3-5p mimics and exosomes groups (*P*  <  0.05), but higher in the miR-550a-a3-5p inhibitor group than in the control group (*P*  <  0.05; Fig. 7A, D–G). Additionally, miR-550a-3-5p inhibition significantly upregulated the expression of these proteins compared with the addition of miR-550a-3-5p mimics (*P*  <  0.05).

### YAP1 directly binds with miR-550a-3-5p

**Fig. 8:**
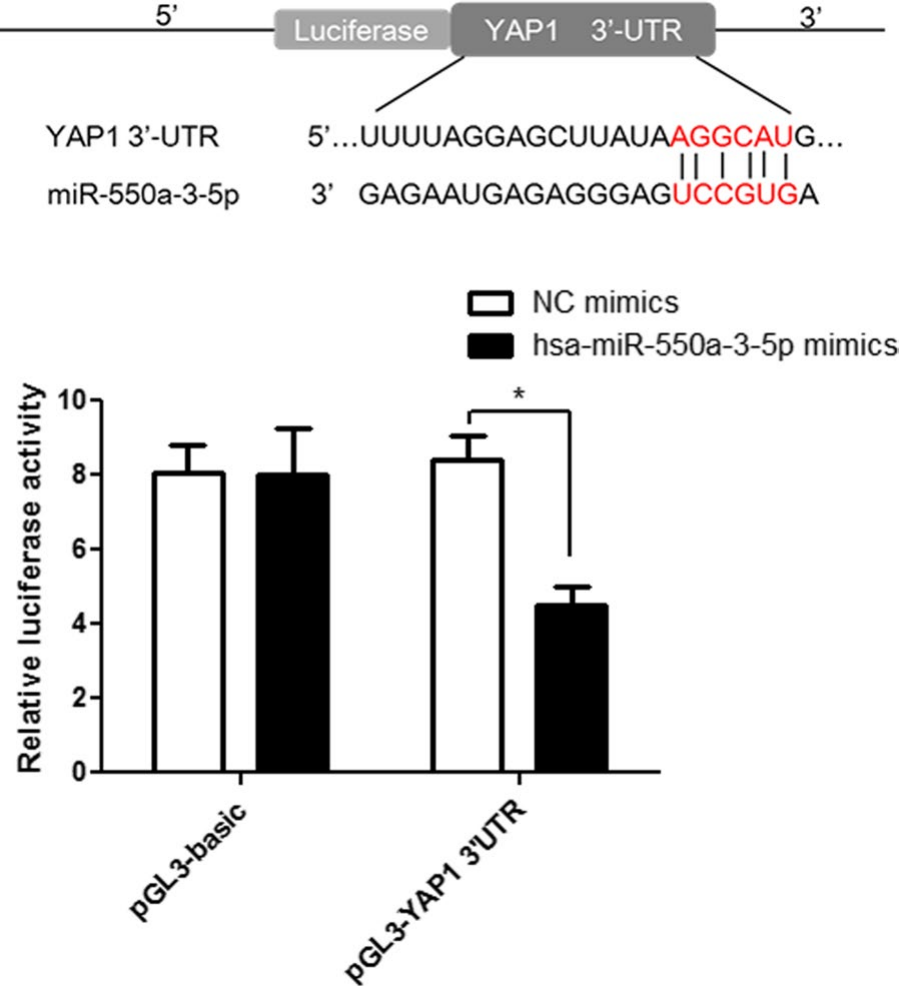
Dual-luciferase reporter gene assay confirmed that YAP1 binds directly with miR-550a-3-5p

*YAP1* was predicted to be the target gene of miR-550a-3-5p in lung cancer cell-derived exosomes, because the 3′-UTR of *YAP1* contains the binding site for miR-550a-3-5p (Fig. 8). To confirm this prediction, a dual-luciferase reporter gene assay was performed. No significant difference in relative luciferase activity was observed between NC mimics and miR-550a-3-5p mimics in the pGL3-basic plasmid (*P * >  0.05). However, relative luciferase activity was significantly decreased following pGL3-YAP1 transfection with miR-550a-3-5p compared with pGL3-YAP1 transfection with NC mimics (*P*  <  0.05; Fig. 8). These results indicate that YAP1 directly binds to miR-550a-3-5p.

## Discussion

Brain metastasis in patients with lung cancer is a major cause of poor prognosis, recurrence, and death [29]. However, there are few reports on the potential molecular mechanisms and therapeutic targets of lung cancer with brain metastasis. Our study identified 22 DE-miRNAs between the exosomes isolated from the plasma of lung cancer patients with or without brain metastasis. RT-qPCR showed that miR-550a-3-5p was significantly enriched in exosomes isolated from lung cancer patients with brain metastasis and miR-550a-3-5p was selected for further cellular experiments. Exosomes were then extracted from low or high metastatic lung cancer cells, and HBMECs were transfected with miRNA NC, miR-550a-3-5p mimics, miR-550a-3-5p inhibitor, and high metastatic lung cancer cell-derived exosomes. It was found that miR-550a-3-5p enrichment, similar with high metastatic lung cancer cell-derived exosomes, inhibited the viability and migration of HBMECs, promoted their apoptosis, and regulated the cell cycle. Western blot analysis results showed that miR-550a-3-5p enrichment significantly upregulated the expression of cleaved-PARP and downregulated the expression of pRB, CDK6, YAP1, CTGF, and CYR61 proteins. Finally, dual-luciferase reporter gene assay indicated that *YAP1* was miR-550a-3-5p’s target gene. Our results identified the miRNAs associated with brain metastasis of lung cancer and indicate that miR-550a-3-5p and YAP1 may be novel potential targets for controlling brain metastasis.

Previous studies have shown that a variety of miRNAs have carcinogenic or tumor suppressive activity and play important roles in the metastasis and progression of lung cancer [30, 31]. In this study, 22 DE-miRNAs (10 upregulated and 12 downregulated) were identified and enriched in 44 BP, 9 CC, and 13 MF, and several signaling pathways, including MAPK, chemokine, PPAR, and Wnt signaling pathways. The MAPK signaling pathway is involved in extensive cellular processes, including viability, proliferation, apoptosis, and differentiation [32]. Zhang et al. [33] demonstrated that the activation of the MAPK signaling pathway is closely associated with the metastasis and invasion of hepatocellular carcinoma. The secretion and production of chemokines such as CCL2, CXCR1/2, CXCL12, CXCR4, and CXCL8 play critical roles in cancer development and subsequent metastasis [34–36]. Aldinucci and Casagrande [37] elaborated on the roles of CCL5 and CCR5 in cell proliferation and metastasis of gastric cancer, and their interaction in regulating immune and inflammatory responses. PPAR has three different subtypes (PPARα, PPARβ/δ, and PPARγ) and has been shown to participate in the brain metastasis of cancer cells [38]. Additionally, the Wnt signaling pathway plays an essential role in maintaining homeostasis and regulating proliferation in various tissues [39]. Nguyen et al. [40] showed that the Wnt/TCF signaling pathway mediates the metastasis of lung adenocarcinoma through HOXB9 and LEF1. Based on the results of our study and those from previous studies, we hypothesize that these 10 upregulated and 12 downregulated DE-miRNAs, together with the MAPK, chemokine, PPAR, and Wnt signaling pathways, may be closely associated with brain metastasis of lung cancer. However, the roles of these pathways in brain metastasis of lung cancer need to be explored further.

Due to the higher level of miR-550a-3-5p in the exosomes isolated from the plasma of lung cancer patients with brain metastasis, miR-550a-3-5p was chosen for further cellular experiments. The results of these experiments showed that miR-550a-3-5p enrichment and high metastatic lung cancer cell-derived exosomes significantly suppressed HBMEC viability and migration, enhanced HBMEC apoptosis, decreased the number of cells during the G0/G1 phase, and increased the number of cells during the S phase, whereas miR-550a-3-5p inhibition had the opposite effects. Wei et al. [41] found that miR-330-3p overexpression promoted the proliferation, migration, and invasion of non-small cell lung cancer (NSCLC) in vitro and facilitated the occurrence of NSCLC tumors in vivo; thus, miR-330-3p may be a biomarker of NSCLC with metastasis. Another study showed that silencing miR-202-3p could destroy the interendothelial junction and enhance the migration of breast cancer cells by upregulating MMP-1, suggesting that miR-202-3p/MMP-1 may be used to predict the metastasis of breast cancer [42]. Together with our results, it can be inferred that exosomal miR-550a-3-5p, as a new biomarker, may affect brain metastasis of lung cancer by regulating the viability, migration, apoptosis, and cycle of HBMECs.

Further, to investigate the underlying molecular mechanisms of miR-550a-3-5p affecting brain metastasis, the expression of cleaved-PARP, pRB, CDK6, YAP1, CTGF, and CYR61 proteins was determined using western blotting. High levels of both miR-550a-3-5p and high metastatic lung cancer cell-derived exosomes markedly upregulated cleaved-PARP protein expression while downregulating pRB, CDK6, YAP1, CTGF, and CYR61 protein expression. PARP plays a key role in detecting genomic damage signals, DNA repair and replication, transcription and regulation of post-transcriptional gene expression, cell growth regulation, and inflammation [43]. PARP is also involved in a variety of pathological processes, including carcinogenesis, regulation of oncogenic transcription factors, and direct or mediated interactions with oncogenes [44]. Zhang et al. [45] showed that lncRNA MALAT1 knockdown could promote the apoptosis of chondrocytes by upregulating cleaved-PARP protein expression, thereby alleviating the progression of osteoarthritis. Previous studies have reported that pRB plays an important role in regulating the expression of target genes that induce cell cycle arrest, apoptosis, and differentiation, and that its dysfunction can contribute to disordered cell growth and chromosomal instability, which are hallmarks of cancer cells [46, 47]. CDK6, as a cell cycle-dependent kinase and a transcriptional regulator, has been reported to lead to disordered cell cycle regulation and uncontrolled cell proliferation, and plays a crucial role in promoting cancer occurrence and development [48, 49]. Zhu et al. [50] showed that let-7c overexpression induced apoptosis in human hepatocellular carcinoma cells and arrested the G1 cell cycle by downregulating the levels of Cyclin D1, CDK6, E2F2, and pRB proteins. CTGF, a member of the CCN stromal cell protein family, participates in many BP, including inducing the expression of inflammatory cytokines, promoting cell adhesion, and regulating tissue remodeling and fibrosis [51, 52]. A previous study indicated that CTGF expression in high bone metastatic prostate cancer cells was 1.9 times higher than in low bone metastatic prostate cancer cells, and the increased CTGF expression did not affect tumor cell proliferation but significantly expanded the tumor area at the site of bone metastasis [53]. CYR61, a secreted matricellular protein, has been shown to accelerate tumor growth, angiogenesis, cell invasion, and metastasis in different solid tumors [54, 55]. Habel et al. [56] demonstrated that CYR61 levels control the expression of markers associated with the process of epithelial-mesenchymal transition, thus inducing tumor cell migration and invasion in osteosarcoma. These findings, combined with our results, support the hypothesis that exosomal miR-550a-3-5p may regulate cell viability, apoptosis, cell cycle, and migration of HBMECs by mediating cleaved-PARP, pRB, CDK6, CTGF, and CYR61 expression, consequently influencing brain metastasis of lung cancer.

In addition, YAP1, as the primary effector of the Hippo pathway, plays an essential role in a variety of biological functions, including cell proliferation, differentiation, and intercellular contact inhibition [57, 58]. Previous studies have shown that abnormal expression of YAP1 occurs in many malignant tumors [58, 59]. Our study found that YAP1 protein expression was significantly downregulated after transfection with miR-550a-3-5p mimics and high metastatic lung cancer cell-derived exosomes, whereas it was significantly upregulated after inhibition of miR-550a-3-5p. Dual-luciferase reporter gene assay results showed that YAP1 directly binds to miR-550a-3-5p. Sun et al. [60] reported that YAP1 was highly expressed in colorectal carcinoma (CRC) tissues, and in vivo and in vitro experiments showed that YAP1 could enhance the proliferation, migration, and invasion of CRC cells. Another study confirmed the negative correlation between miR-550a-3-5p and YAP1 in colon cancer tissues, and showed that miR-550a-3-5p could repress tumor cell proliferation, metastasis, and sphere formation by directly inhibiting YAP1 and its oncogenic pathway in various cancer cell types [27]. Taken together, exosomal miR-550a-3-5p may control the progression of lung cancer with brain metastasis by targeting YAP1 expression.

## Conclusions

In conclusion, through exosome sequencing, we identified 22 DE-miRNAs, and showed that the MAPK, chemokine, PPAR, and Wnt signaling pathways may be associated with brain metastasis of lung cancer. Additionally, *YAP1* was identified as the target gene of exosomal miR-550a-3-5p, and miR-550a-3-5p may regulate the growth and migration of HBMECs by mediating YAP1, cleaved-PARP, pRB, CDK6, CTGF, and CYR61 protein expression, thus controlling brain metastasis of lung cancer. However, further experiments using additional clinical samples need to be carried out to verify these results. Our findings will help to improve our understanding of the progression of brain metastasis and provide a basis for using miR-550a-3-5p and YAP1 as potential targets for controlling brain metastasis of lung cancer.

## Supplementary Information

**Additional file 1: Figure S1.** Functional analyses of differentially expressed differentially expressed miRNAs (DE-miRNAs). (A) Gene Ontology terms analysis of DE-miRNAs. (B) Kyoto Encyclopedia of Genes and Genomes pathways enrichment of DE-miRNAs.

## Data Availability

The dataset used and/or analyzed during the current study are available from the corresponding author on reasonable request.
