# Supplementary material for: MicroRNA-550a-3-5p controls the brain metastasis of lung cancer by directly targeting YAP1
Source: Cancer Cell Int. 2021 Sep 16;21:491. doi: 10.1186/s12935-021-02197-z (PMC8444378; doi:10.1186/s12935-021-02197-z)
Supplement: Supplementary file 1 — Additional file 1: Figure S1. Functional analyses of differentially expressed differentially expressed miRNAs (DE-miRNAs). (A) Gene Ontology terms analysis of DE-miRNAs. (B) Kyoto Encyclopedia of Genes and Genomes pathways enrichment of DE-miRNAs. [file 12935_2021_2197_MOESM1_ESM.docx]

**
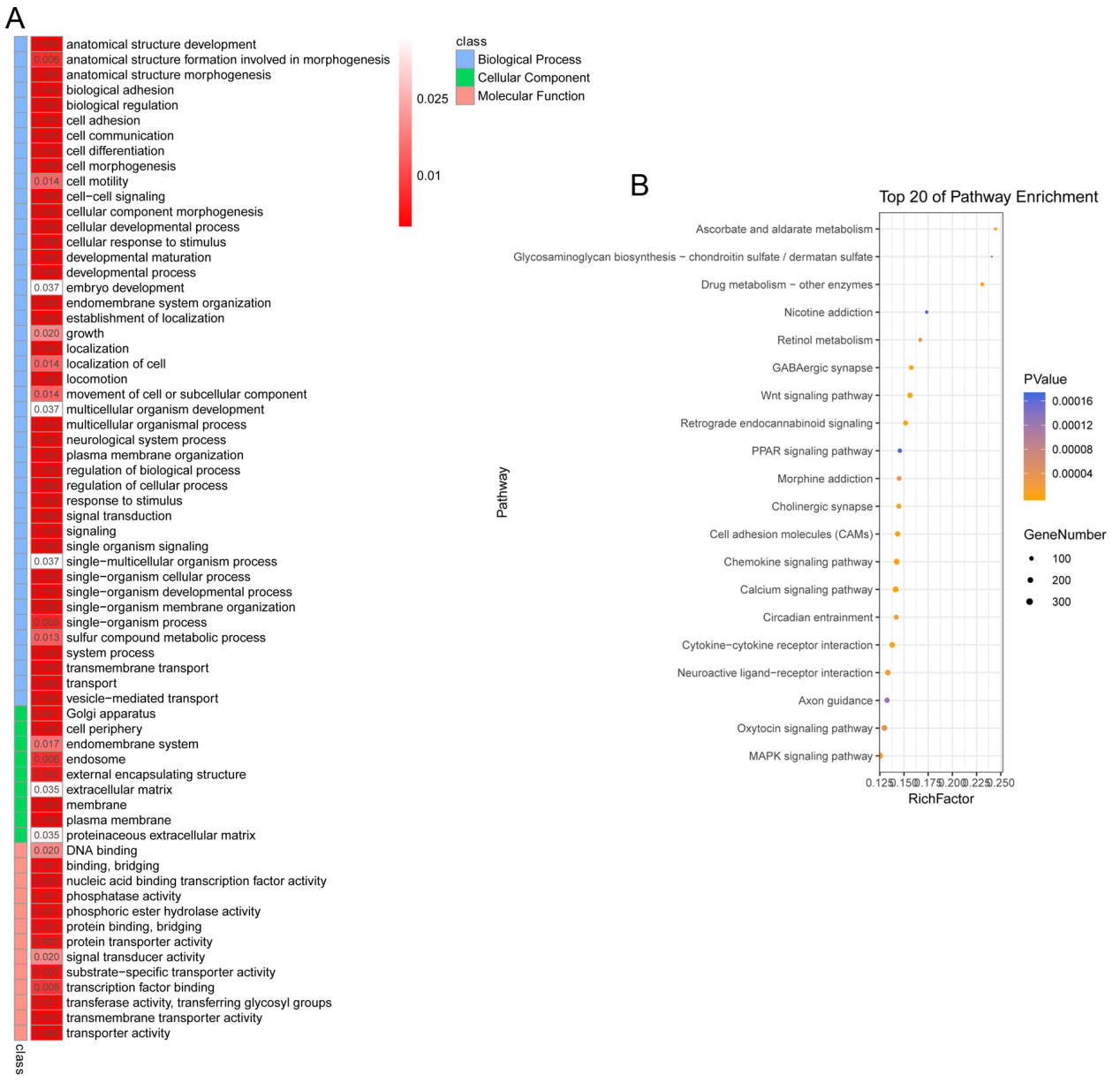
**

**Supplementary figure 1**

Functional analyses of differentially expressed differentially expressed miRNAs (DE-miRNAs). (A) Gene Ontology terms analysis of DE-miRNAs. (B) Kyoto Encyclopedia of Genes and Genomes pathways enrichment of DE-miRNAs.
